# Long-range exciton diffusion in molecular non-fullerene acceptors

**DOI:** 10.1038/s41467-020-19029-9

**Published:** 2020-10-15

**Authors:** Yuliar Firdaus, Vincent M. Le Corre, Safakath Karuthedath, Wenlan Liu, Anastasia Markina, Wentao Huang, Shirsopratim Chattopadhyay, Masrur Morshed Nahid, Mohamad I. Nugraha, Yuanbao Lin, Akmaral Seitkhan, Aniruddha Basu, Weimin Zhang, Iain McCulloch, Harald Ade, John Labram, Frédéric Laquai, Denis Andrienko, L. Jan Anton Koster, Thomas D. Anthopoulos

**Affiliations:** 1 King Abdullah University of Science and Technology (KAUST), KAUST Solar Center (KSC), Physical Sciences and Engineering Division (PSE), Material Science and Engineering Program (MSE), 23955-6900 Thuwal, Kingdom of Saudi Arabia; 2 University of Groningen, Zernike Institute for Advanced Materials, Nijenborgh 4, 9747 AG Groningen, The Netherlands; 3 Max Planck Institute for Polymer Research, Ackermannweg 10, 55128 Mainz, Germany; 4 Department of Physics, Imperial College London, South Kensington, London, SW7 2AZ UK; 5 Electrical Engineering and Computer Science, Oregon State University, 3103 Kelley Engineering Center, Corvallis, OR 97331 USA; 6 Department of Physics, Organic and Carbon Electronics Laboratories (ORaCEL), North Carolina State University, Raleigh, NC 27695 USA

**Keywords:** Solar cells, Electronic devices, Solar cells

## Abstract

The short exciton diffusion length associated with most classical organic semiconductors used in organic photovoltaics (5-20 nm) imposes severe limits on the maximum size of the donor and acceptor domains within the photoactive layer of the cell. Identifying materials that are able to transport excitons over longer distances can help advancing our understanding and lead to solar cells with higher efficiency. Here, we measure the exciton diffusion length in a wide range of nonfullerene acceptor molecules using two different experimental techniques based on photocurrent and ultrafast spectroscopy measurements. The acceptors exhibit balanced ambipolar charge transport and surprisingly long exciton diffusion lengths in the range of 20 to 47 nm. With the aid of quantum-chemical calculations, we are able to rationalize the exciton dynamics and draw basic chemical design rules, particularly on the importance of the end-group substituent on the crystal packing of nonfullerene acceptors.

## Abstract (web summary)

The short-range diffusion length of organic semiconductors severely limits exciton harvesting and charge generation in organic bulk heterojunction solar cells. Here, the authors report exciton diffusion length in the range of 20 to 47 nm for a wide range of non-fullerene acceptors molecules.

## Introduction

After a few years of stagnation in terms of efficiency, organic solar cells (OSCs) are back in the spotlight thanks to the advent of new non-fullerene acceptor (NFA) molecules^1–3^. NFAs have brought OSCs’ power conversion efficiency (PCE) to new heights with records set between 16–18.2% for single-junction^4–11^ and 15–17.3% for tandem cells^12–16^. While recent progress has been impressive, the aforementioned levels of performance are still below the predicted efficiency limit of 20% and 25% for single-junction and tandem cells, respectively^17,18^. Recent efforts towards increasing the PCE of OSCs have been motivated by research on new materials with improved charge carrier mobility and broader spectral absorption^2,3,9^. However, it is important that exciton formation, dissociation, and subsequent charge collection efficiencies are all simultaneously maximized, yielding the highest possible photocurrent.

In OSCs, successful absorption of a photon generates an exciton, a coulombically bound electron–hole pair^19^. Thermal dissociation of excitons within a low dielectric medium such as an organic semiconductor is highly improbable. To efficiently generate free charges, two semiconductors with suitable energetics, an electron donor and electron acceptor are intermixed, forming a so-called bulk-heterojunction (BHJ) cell. One of the prerequisites for efficient exciton harvesting is the fast exciton diffusion to the donor–acceptor interface, where it dissociates into free charges. The diffusion constant and the exciton lifetime set the optimal size of the donor and acceptor domains within the BHJ. Up until now, most of the work on exciton diffusion length in OSCs has been focused on electron–donor (*p*-type) materials^20^ with very few reports on molecular NFAs^21,22^. Recent work has shown that fused-ring acceptors such as indacenodithiophene end-capped with 1,1-dicyanomethylene-3-indanone (IDIC) exhibit long exciton diffusion length with a diffusion constant of at least 0.02 cm^2^/s^21^. This is consistent with the large domain sizes of 20–50 nm often reported for high-efficiency NFA-based BHJ cells^8,23–26^. It is not yet fully understood why the exciton diffusion length in NFAs, such as IDIC, is significantly higher than in amorphous and other polycrystalline organic semiconductors (typically 5–20 nm)^20^.

Here, we study the exciton diffusion length (*L*_D_) in a wide range of NFAs^1–3,27–31^ (chemical names of all materials can be found in “Methods” section) using two independent experimental techniques, one relying on copper(I) thiocyanate (CuSCN)/NFA bilayer OPV measurements and the other on exciton annihilation spectroscopy. We focus on best-in-class acceptor–donor–acceptor (A–D–A) NFAs comprising different end-groups ranging from methyl (IT-M) to chlorine (IT-2Cl) and fluorine (IT-4F), including the current PCE record holder Y6. The measured *L*_D_ is found to vary with IT-4F, amongst all NFAs studied, exhibiting the longest diffusion length of 45 nm. This value is >4 times higher than the ≈10 nm reported for the prototypical fullerene-based acceptor PC_71_BM. To elucidate the origin of the long *L*_D_, we combine crystallographic data with quantum-chemical calculations for each molecule. The simulations predict distinctly large excitonic couplings due to aligned transition dipole moments in the crystal, relatively small reorganization energies due to the stiff conjugated core, and quadrupolar symmetry of the excitation—i.e. small energetic disorder—the combination of which leads to the large exciton diffusion lengths observed, in good agreement with our simulations. Key relationships between *L*_D_ and the chemical structure of the NFA are identified, leading to important design guidelines for future generation NFAs.

## Results

### Material properties

**Fig. 1:**
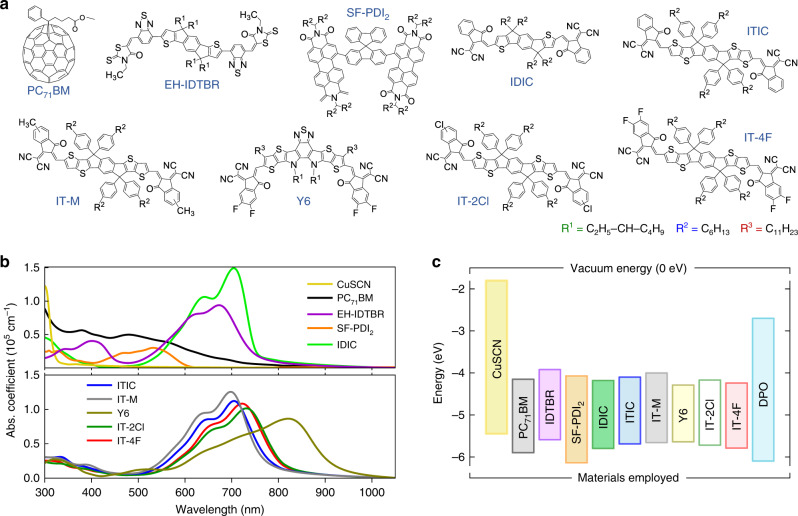
Molecular structure, absorption, and energy levels of the organic acceptors studied. **a** Chemical structure of the acceptors investigated in this study. Full names are provided in the “Methods” section. **b** Absorption spectra of the acceptor molecules studied. **c** The IE values obtained from PESA measurements (see Supplementary Fig. 1) and IE + optical gap (*E*_g_), *E*_g_ were estimated from the intersection of the UV–Vis absorption spectra and photoluminescence spectra. DPO is a short name for an electron extraction layer of Phen-NaDPO ((2‐(1,10‐phenanthrolin‐3‐yl)naphth‐6‐yl)diphenylphosphine oxide). The IE value of DPO was obtained from our previous work^68^.

Figure 1a illustrates the chemical structures of the acceptor materials studied, while Fig. 1b shows their absorption spectra together with that of the hole-transport layer CuSCN. The latter is a known wide bandgap (>3.4 eV) inorganic *p*-type semiconductor absorbing only in the ultraviolet region^32,33^. With the exception of SF-PDI_2_, all other NFAs absorb across the visible (Vis) all the way to the near-infrared, while exhibiting higher absorption coefficients than PC_71_BM. Figure 1c depicts the ionization energies (IE) of the acceptors and the carrier-transport layers as determined by photoelectron spectroscopy in air (PESA).

### Exciton diffusion length calculation using bilayer CuSCN/acceptor devices

**Fig. 2:**
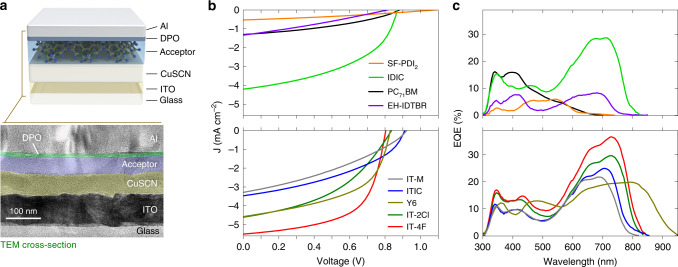
Device performance and EQE spectra of the bilayer CuSCN/acceptor devices. **a** Schematic of the device architecture and the cross-sectional transmission electron microscopy (TEM) image of a CuSCN/acceptor bilayer solar cell. **b** Current density–voltage (*J*–*V*) characteristics of OPV cells measured under simulated solar illumination. **c** Corresponding EQE spectra of the bilayer OPVs shown in **b**.

Figure 2a illustrates the structure and energy diagram of a planar CuSCN/acceptor heterojunction solar cell used to study the *L*_D_. The use of CuSCN allows for efficient extraction of the photogenerated holes while simultaneously blocking electrons, effectively acting as exciton quencher for the organic absorber. Efficient exciton quenchers for *n*-type organic semiconductor, such as NFAs, are scarce and the particular CuSCN/*n*-type semiconductor platform for exciton diffusion studies has not been reported before. As electron extracting layer, we employed the wide bandgap Phen-NaDPO (DPO) ((2‐(1,10‐phenanthrolin‐3‐yl)naphth‐6‐yl)diphenylphosphine oxide)^34^. Adjusting the thickness of the organic semiconductor and measuring the OPV performance allows the study of *L*_D_ within the organic layer, without the morphology-related complexities encountered in organic BHJs^35^.

To estimate *L*_D_ within the acceptor layer, we used a similar method to that described by Siegmund et al.^35^. The external quantum efficiency (EQE) of the bilayer cells is measured as a function of acceptor thickness, and then the measured photocurrent is modeled using the exciton diffusion equation:

1 $$\frac{{\partial n}}{{\partial t}} = D\frac{{\partial ^2n}}{{\partial x^2}} + G\left( {x,t} \right) - k_{{\mathrm{PL}}}n - k_{{\mathrm{FRET}}}n - \alpha n^2$$

where *n* is the singlet exciton density at position *x* in the absorber film, *D* is the diffusion coefficient, *G*(*x*, *t*) is the time-dependent exciton generation profile, *k*_PL_ is the radiative decay rate in absence of quencher sites, *α* is an exciton–exciton annihilation rate constant, and *k*_FRET_ denotes the rate of Förster resonance energy transfer (FRET) in the presence of a neighboring material.

To accurately measure *L*_D_ of the NFAs with the bilayer cell, it is important to ensure that: (i) the exciton dissociation happens only at a well-defined quencher–acceptor interface^36^; (ii) the generation of exciton originates only from the acceptor; and (iii) the photocurrent is not limited by the transport properties of the NFA layer.

Figure 2a shows the schematic of the device and a representative cross-sectional transmission electron microscopy (TEM) image of a CuSCN/NFA bilayer cell. Well-defined interfaces are visible across the device ensuring that requirement (i) is satisfied. Figure 2b shows representative *J*–*V* curves for the bilayer CuSCN/NFA cells. Devices with IT-4F show a maximum PCE of 2.65% and *J*_SC_ > 5 mA cm^−2^ (Supplementary Table 1). The EQE spectra of the devices (Fig. 2c) reveal that charge generation occurs across the entire acceptors’ absorption spectra range while CuSCN does not contribute to the generation of excitons (Fig. 1b), hence satisfying requirement (ii). The requirement (iii) is also fulfilled as the performance of the bilayer solar cells is not limited by the charge transport of the acceptor materials as evident from the sufficiently high and ambipolar mobility values extracted using thin-film transistors (TFTs) (Supplementary Fig. 2) and time-resolved microwave conductivity (TRMC) measurements (Supplementary Table 2). The sufficiently high mobility is due to the NFAs investigated in this work exhibit a certain degree of crystallinity as shown from grazing incident wide-angle X-ray scattering (GIWAXS) measurements (Supplementary Fig. 3).

CuSCN is particularly suitable as an exciton quencher for this type of measurement, since FRET from CuSCN to the acceptor layer is negligible due to the very low fluorescence of CuSCN and the small overlap of its absorption with the acceptors’ emission (Supplementary Fig. 4). Exciton–exciton annihilation (*α*) is also negligible at the considered intensities. As a result, the dominant exciton harvesting mechanism within the acceptor layer is exciton diffusion. Hence, Eq. (1) can be simplified under steady-state conditions with *k*_PL_ = *D*/*L*_D_^2^ such as

2 $$\left( {\frac{{\partial ^2}}{{\partial x^2}} - \frac{1}{{L_{\mathrm{D}}^2}}} \right)n(x) = \frac{{G\left( x \right)}}{D}$$

which can be solved for any generation assuming that the excitons are fully quenched at the interface *n*(interface) = 0. The EQE can then be calculated considering that the photocurrent is only due to the exciton dissociation at the CuSCN/NFA interface:

3 $${\rm{EQE}} = \frac{{J_{{\mathrm{photo}}}}}{{J_{{\mathrm{inc}}}}} = \left. {\frac{{q\eta _{\mathrm{c}}D}}{{J_{\rm{{{inc}}}}}}\frac{{\partial n(x)}}{{\partial x}}} \right|_{{\mathrm{interface}}}$$

where *J*_photo_ and *J*_inc_ are the generated photocurrent density and the incident light current density, *η*_c_ the combination of the exciton dissociation and extraction at the electrode efficiencies^35^.

There are four main factors that influence the magnitudes of *J*_photo_ and EQE, namely the absorption coefficient, *η*_c_, *D* and *L*_D_ (Supplementary Fig. 5a, b). The contribution of the absorption is included in the generation profile *G*(*x,λ*_exc_), evaluated using transfer-matrix modeling^37^. However, *η*_c_ and *D* have no influence on the shape of the EQE vs. thickness curve, only on its absolute value. As a result, we can only obtain the *D* × *η*_c_ product and as such we will not discuss those values at this point. Instead, we focus on fitting Eqs. (2) and (3) to normalized EQE vs. thickness data, as it is mostly influenced by *L*_D_ hence allows reliable estimation of its magnitude^35^. The fits assumed that geminate and nongeminate recombination losses are independent of thickness which is valid in our case as confirmed by light-intensity and thickness dependent of the *J*–*V* characteristics of IT-4F devices (Supplementary Fig. 5c, d). We also coupled the results from solving Eq. (2) with drift-diffusion simulation^38^ (Supplementary Fig. 5e, f, Supplementary Table 3) to demonstrate that the photocurrent measurement is not limited by the mobility of the NFAs or the recombination. However, there could be cases (e.g. extremely thick layers or very low mobility materials) where recombination losses do depend on layer thickness which can lead to incorrect values of the exciton diffusion length.

**Fig. 3:**
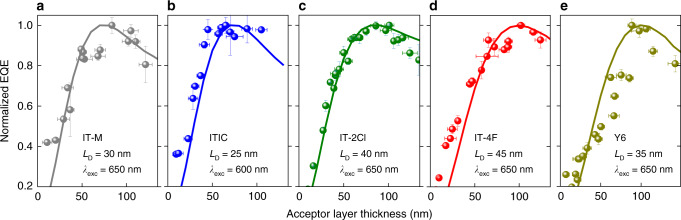
Exciton diffusion length estimated from the EQE spectra of bilayer CuSCN/NFA devices. EQE spectra of CuSCN/NFA bilayer devices for different NFA layer thickness. **a** IT-M, **b** ITIC, **c** IT-2Cl, **d** IT-4F, and **e** Y6, measured using an excitation wavelength of *λ*_exc_ = 650 nm (600 nm for ITIC). The experimental data (circles) are fitted (solid lines) for all NFA thicknesses.

To estimate *L*_D_, we measured the EQE spectra of the bilayer devices with different NFA layer thicknesses, whilst maintaining the thickness of the CuSCN layer to ca. 60 nm. As shown in Fig. 3, the measured EQE (at *λ*_exc_ = 650 nm, see Supplementary Figs. 6–14 for EQE at different *λ*_exc_) reaches a maximum value for acceptor thickness between 60 and 100 nm, which indicates long *L*_D_ values. Analysis of the data yields an exciton diffusion length in IT-M and ITIC of *L*_D_ = 25–30 nm. Incorporating electron-deficient elements like F or Cl into the end-capping groups, such as in the case of IT-2Cl and IT-4F acceptors, results in an increase of *L*_D_ to 40–45 nm. For the remaining acceptors, the *L*_D_ values are summarized in Table 1. For the Y6 acceptor, with recently reported PCE values reaching in the range 15–18.2% when blended with best-in-class donor polymers^3,5–7,9,10^, we calculate an *L*_D_ value of 35 nm. Additionally, we obtained *L*_D_ ≈ 10 nm for PC_71_BM, which is close to that of C_70_ (7.4 nm) but significantly smaller than that of C_60_ (18.5 nm) obtained using a photocurrent method^36^. For comparison, the exciton diffusion length of PC_71_BM obtained by a different measurement technique, namely PL quenching in blends, was reported to be 3.1 or 4.5 nm^39,40^. The constraint of this technique is, however, that the quencher must be intimately mixed with the matrix material, since demixing in the blend does lead to lower diffusion coefficients as the quencher concentration is increased^20^.

**Table 1:** Summary of diffusion length (*L*_D_) values for all NFA materials studied as well as spectral overlap *J* and Förster radius *R*_0_.

Acceptor	*L*_D,EQE_ (nm)	*L*_D,TA_ (nm)	*D* (10^-2^ cm^2^ s^-1^)	*τ* (ps)	*J* (10^30^ nm^6^ mol^-1^)	*R*_0_ (nm)
PC_71_BM	10	–	–	–	0.15	1.4
EH-IDTBR	15	–	–	–	1.11	3.2
SF-PDI_2_	20	–	–	–	0.02	2.7
IDIC	24	16.4 ± 0.3	1.11 ± 0.01	241 ± 8	1.00	2.8
ITIC	25	31.9 ± 0.7	2.59 ± 0.06	394 ± 15	1.94	3.3
IT-M	30	34.9 ± 0.6	3.10 ± 0.08	392 ± 8	1.90	3.1
Y6	35	37.0 ± 1.1	5.41 ± 0.05	253 ± 15	3.92	2.8
IT-2Cl	40	41.4 ± 0.5	4.26 ± 0.03	402 ± 9	2.39	2.9
IT-4F	45	47.4 ± 0.9	6.41 ± 0.08	351 ± 12	2.30	2.8

*L*_D_ obtained using photocurrent and transient absorption (TA) techniques. Diffusion constant (*D*) inferred from the TA measurements and was calculated by assuming the annihilation radius of singlet excitons to be 1 nm.

The fits reproduce well the experimental data (Supplementary Figs. 6–14) and the values of *L*_D_ are estimated based on the sensitivity of the fit over a range of thickness between 10 and 150 nm for at least four different excitation wavelengths. The accuracy of these fits depends on the experimental uncertainties of the EQE, the layer thicknesses, and the values of the complex refractive index. The variation in refractive index may explain the deviation from the fit for thin NFA layers^35^. We find that decent fits can be obtained, for most of the NFAs, when varying *L*_D_ within a margin of ≈5–10 nm (Supplementary Figs. 6–14).

### Exciton diffusion length measured via exciton annihilation

We independently validated the photocurrent-based measurements of *L*_D_, using the exciton annihilation method^21^. The latter technique uses ultrafast transient absorption (TA) spectroscopy to measure exciton lifetimes as a function of excitation density. The measurement is carried out on bulk films and does not require exciton quenching interfaces. When exciton annihilation occurs in the film, the exciton decay is accelerated with increasing excitation fluence. The excitation fluences used here range from 0.3 to 11 μJ cm^−2^. The exciton decay can be globally fit to a rate equation accounting for exciton annihilation and first-order recombination of the excitons^21,41^:

4 $$\frac{{{\mathrm{d}}n\left( t \right)}}{{{\mathrm{d}}t}} = \kappa n\left( t \right) - \frac{1}{2}\alpha n^2(t)$$

which leads to the following solution:

5 $$n\left( t \right) = \frac{{n(0)e^{ - \kappa t}}}{{1 + \frac{\alpha }{{2\kappa }}n\left( 0 \right)[1 - e^{ - \kappa t}]}}$$

Here, *κ* is the fluorescence decay rate constant in the absence of any annihilation, *α* is the singlet–singlet bimolecular exciton annihilation rate constant, *n*(*t*) is the singlet exciton density as a function of time after the laser excitation. The measurement requires two sets of films prepared on a quartz glass substrate: (1) neat acceptor film to obtain the *α* value and (2) dilute acceptor in polystyrene film to extract *κ* (or intrinsic exciton lifetime, *τ* = 1/*κ*).

**Fig. 4:**
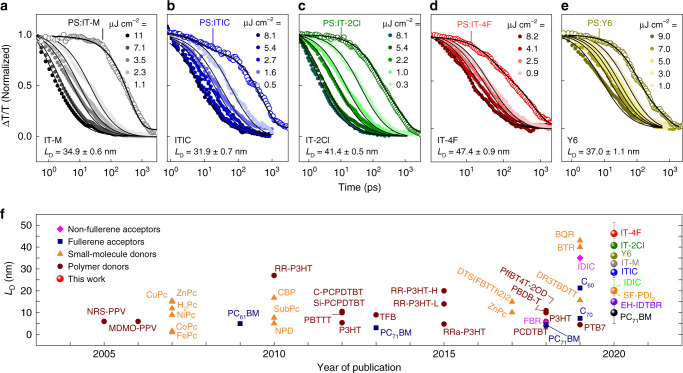
Exciton diffusion lengths of various NFAs estimated from singlet-singlet annihilation measurements and non-exhaustive comparison of diffusion length of organic donor and acceptor materials. Singlet–singlet exciton annihilation decay in neat films of: **a** IT-M (excitation wavelength: 700 nm), **b** ITIC (700 nm), **c** IT-2Cl (700 nm), **d** IT-4F (750 nm), and **e** Y6 (700 nm). Fluence-dependent singlet exciton decays of the neat films fitted to the exciton annihilation model (Eq. (4)). The exciton decays of the NFAs diluted in a polystyrene (PS) matrix are also superimposed in this figure. **f** Comparison of diffusion lengths reported from 2005 onwards for various types of donor and acceptor materials relevant to organic photovoltaics. Publication details can be found in Supplementary Table 5.

Figure 4a–e shows the TA data for IT-M, ITIC, IT-2Cl, IT-4F, and Y6, all of which indicate a clear sign of exciton annihilation, where the exciton decay is substantially accelerated with increasing excitation fluence. The exciton decays for all the films can be fitted with Eq. (5), with the only free parameter being the bimolecular rate constant (*α*). The exciton diffusion length can be calculated as *L*_D_ = (*Dτ*)^1/2^, where *D* is the diffusion constant given by *D* = *α*/(8*πR*) (three-dimensional diffusion model), *R* is the annihilation radius of singlet excitons. The annihilation radius cannot be easily measured and generally assumed to be 1 nm^21,41–43^. In the case of the small molecule donor DTS(FBTTh_2_)_2_, *R* has been measured to be around 1.1 nm^44^. The latter has also been assumed to be equal to the intermolecular distance which can be obtained from x-ray diffraction studies (~1 nm for DPP-based small molecule donors^45^) or *d*_100_ spacing obtained from GIWAXS of neat films (1.7–1.9 nm for BTR and BQR small molecule donors^46^). Here, we used two different values of annihilation radius (*R* = 1 nm and *R* equal to  *d*_100_ spacing of the NFAs, Supplementary Table 4). When *R* = 1 nm was used in our calculation, a remarkable agreement between the *L*_D_ values obtained from singlet–singlet annihilation (SSA) analyses (Fig. 4) with those derived from the photocurrent measurements (Table 1), is evident. Notably, the *L*_D_ value of 47 nm measured for IT-4F using SSA analyses is very close to *L*_D_ = 45 nm inferred from the photocurrent method (Fig. 3d). The SSA analyses also yield *D* = 0.064 cm^2^ s^−1^ which is two times higher than IT-M and ITIC. The similarity in the exciton diffusion values measured between thin films and bilayer solar cells suggests that the values in Table 1 represent the intrinsic *L*_D_. In Fig. 4f we compare the exciton diffusion values reported in the literature with those extracted here. Evidently, the NFAs studied here exhibit the highest *L*_D_ amongst the OSCs materials (details in Supplementary Table 5). Longer *L*_D_ values have only been reported for conjugated polymer nanofibers (>200 nm)^47^, small molecule J-aggregates (96 nm)^48^, or organic single-crystals (>1 μm)^49,50^.

### Energetic disorder and Förster transfer radius

We performed temperature-dependent (150–300 K), steady-state PL measurements on all acceptor materials to probe the energetic disorder (Supplementary Fig. 15). The energetic disorder of the exciton density of states (DOS), *σ*, was calculated from the slope of the 0–0 peak position (*E*_0–0_) vs. 1/*T* (see Supplementary Table 6) following previous works^20,21^. The calculation assumes that the exciton relaxes toward tail states of the DOS and eventually settles in the occupied density of states (ODOS) at a mean energy ‒*σ*^2^/*kT* below the center of the DOS^20^. For EH-IDTBR, SF-PDI_2_, ITIC, IT-M, and Y6, two temperature regimes can be identified, one at higher (220–310 K) and one at lower (140-220 K) temperature range (Supplementary Fig. 15). At higher temperatures, *E*_0–0_ exhibits a relatively strong dependence on temperature but weakens for *T* < 220 K. This is consistent with the activated diffusion model observed in previous works^21,51^. In contrast, IT-Cl, IT-4F, and IDIC exhibit a single temperature regime (Supplementary Fig. 15) with lower *σ* values of 45, 39, and 34 meV, respectively. Comparable *σ* values in the range of 10–23 meV have recently been reported for IDIC^21^. Using photothermal deflection spectroscopy (PDS), we found the energetic disorder in IDTA and IDTTA NFA films to be between 24 and 28 meV^14^. For comparison, a *σ* value of 44 meV was reported for the conjugated polymer MDMO–PPV^51^ and 39 meV for P3HT^52^. A very low disorder width of 15 meV has been observed in structurally rigid molecules such as the ladder-type-conjugated chromophore^53^.

We attempted to rationalize the exciton diffusion length using a FRET model. The rate *k*_F_ of FRET between chromophores is given by^20^

6 $$k_{\mathrm{F}}\left( d \right) = \frac{1}{{\tau _0}}\left( {\frac{{R_0}}{d}} \right)^6$$

where *τ*_0_ is the intrinsic exciton lifetime that is not limited by diffusion quenching at defects, *d* is the distance between chromophores, and *R*_0_ is the Förster radius written as^20^

7 $$R_0^6 = \frac{{9\kappa ^2\varphi _{{\mathrm{PL}}}J}}{{128\pi ^5N_{\mathrm{A}}n^4}}$$

Here *φ*_PL_ is photoluminescence (PL) quantum yield, *κ* is the dipole–dipole orientation factor and here we use *κ*^2^ = 0.476 corresponding to static and randomly oriented dipoles^45,54^, *n* is the refractive index, *N*_A_ is Avogadro number, and *J* is the spectral overlap for energy transfer between chromophores ($J = \smallint \varepsilon \left( {\lambda _{\mathrm{s}}} \right)f\left( {\lambda _{\mathrm{s}}} \right)\lambda _{\mathrm{s}}^{ - 4}{\rm{d}}\lambda _{\mathrm{s}}$). The integral *J* over wavelength, $\lambda _{\mathrm{s}}$, quantifies the spectral overlap between the area-normalized PL spectrum of donor ($f\left( {\lambda _{\mathrm{s}}} \right)$) and the absorption spectrum of acceptor expressed in terms of molar-absorptivity ($\varepsilon \left( {\lambda _{\mathrm{s}}} \right)$).

Supplementary Fig. 16 shows the absorption and PL spectra of all acceptor molecules studied. All A–D–A NFAs exhibit high absorption coefficients and large overlap with the PL spectra resulting in a *J* value in the range of 1–4 × 10^16^ nm^4^ M^−1^ cm^−1^ (Table 1). The large overlap between absorption and emission indicates small intramolecular reorganization (i.e. small barrier for the exciton transfer between chromophores) and yields Förster transfer radius between 2.6 and 3.2 nm (Table 1). This is in agreement with the previous report on IDIC, where facile exciton diffusion results from many FRET parameters being optimized for long-range transport^21^. In comparison, PC_71_BM shows one order of magnitude lower spectral overlap integral *J* (1.5 × 10^15^ nm^4^ M^−1^ cm^−1^) that gives *R*_0_ = 1.3 nm. On the other hand, *R*_0_ for perylene diimide-based NFA (SF-PDI_2_) is large (2.6 nm) despite the very low spectral overlap (2 × 10^14^ nm^4^ M^−1^ cm^−1^), due to the much higher *ϕ*_PL_ (Supplementary Table 6). While the difference in *R*_0_ explains the difference in diffusion length between PC_71_BM and the NFAs, it cannot explain the variation between the NFAs (Supplementary Fig. 17).

### Quantum chemical calculations

Therefore, we turn to quantum chemical calculations to correlate the main chemical structure and the end-group substituents with the exciton diffusion length and diffusion coefficient. Based on Fermi’s Golden rule, the energy transfer rate can be written as $\nu = \frac{{2{\uppi}}}{\hbar }V^2J$, where $V$ is the electronic coupling element, and $J$ is the Franck–Condon weighted DOS which is normally approximated by the spectral overlap of the donor emission and of the acceptor absorption^55^. Instead of explicitly calculating the spectral overall of Franck–Condon factors, we assume that it is related to the molecular reorganization energy, *λ*, i.e., adapt a harmonic approximation for the promoting mode. In this case $\nu = \frac{{V^2}}{\hbar }\sqrt {\frac{{\uppi }}{{\lambda {{k}}_{\mathrm{B}}T}}} \exp \left( { - \frac{\lambda }{{4k_{\mathrm{B}}T}}} \right)$ is given by the classical Marcus rate which, despite all imperfections, can be used for qualitative analysis of exciton transport^56^.

**Fig. 5:**
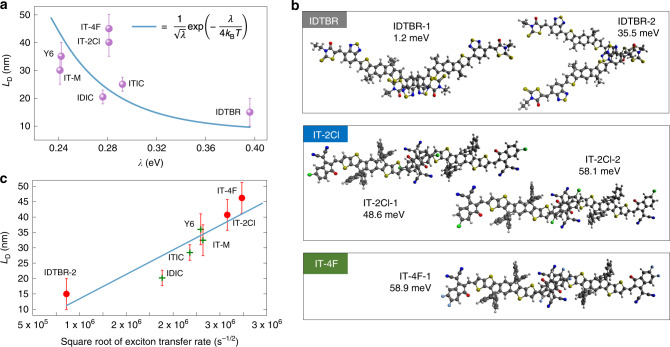
Quantum chemical calculations of reorganization energy and the exciton transfer rate of NFAs. **a** The correlation of the measured diffusion length and the reorganization energy, with a fixed intramolecular excitonic coupling. The dashed line corresponds to the Marcus rate. **b** Featured dimer structures and the corresponding exciton coupling parameters of IDTBR, IT-2Cl, and IT-4F. **c** Correlation between the measured exciton diffusion length and calculated square root of exciton transfer rate. The line is the guide to the eye.

Reorganization energies were calculated at the m06-2×/6-311g(d,p) level using the Gaussian16 package^57^ and are listed, together with the oscillator strengths at the optimized ground state and first excited state geometries in Supplementary Table 7. Substituting these reorganization energies into the Marcus rate and assuming a constant electronic coupling element, we obtain a reasonable correlation between the rate $\nu$ and *L*_D_ (Fig. 5a). There are, however, a few outliers, in particular, the ITIC family has similar reorganization energies but different *L*_D_. To explain this we have calculated the excitonic coupling *V* using the diabatization scheme which maximizes the contribution of one single excitation configuration to an excited state (using eight excited states for constructing diabatic states, Supplementary Table 8)^58^. For IDTBR and IT-2Cl crystals, we extracted two types of ${\uppi}$-stacked dimers, while for IT-4F there is only one type. The dimers and the calculated exciton couplings are shown in Fig. 5b. For NFAs with unresolved crystal structures, we have used an average coupling of 40 meV. Figure 5c shows the calculated exciton transfer rates plotted versus the measured *L*_D_ where a clear correlation can be seen. We can, therefore, conclude that a significant boost to the rate is due to the stiffening of the conjugated core, from IDTBR to ITIC to Y6, which reduces the activation barrier for the exciton transfer. For a given reorganization energy, the rate is then further enhanced by the crystalline packing, such as the one achieved in the crystal of IT-4F.

Another interesting observation is that a large coupling between the donor and acceptor blocks of the acceptor–donor–acceptor-conjugated core leads to a quadrupolar-type excitation, with both acceptor blocks having an excess electron upon excitation^59–61^. The immediate implication is that the excited state does not have a dipole, but a quadrupole moment. Hence, the interaction of the excited molecule with the fluctuating fields created by the neighboring molecules in a film is of a quadrupole–quadrupole, not a dipole–quadrupole type. This reduces both energetic disorder (larger diffusion length) and external reorganization energy (larger exciton transfer rates), all thanks to the conjugated A–D–A molecular architecture.

To confirm this, we have calculated energetic disorder of excited states for materials with crystal structures available experimentally (IDTBR, IEICO-4F, IT-4F, ITIC). The DOS shown in Supplementary Fig. 18a confirms that the energetic disorder of all compounds is about 50 meV, in agreement with experimental data (Supplementary Table 6). Narrow DOS naturally boosts the exciton mobility (no trapping). In the case of ITIC, we observe several peaks (the green and blue curves in Supplementary Fig. 18a), which is due to two different types of molecular orientations in the crystal packing, see Supplementary Fig. 18b, contrary to IDTBR/IEICO-4F, where molecular environments for different molecules are similar, leading to a uniform peak broadening. Hence, the two slopes of the linear dependence (see Supplementary Fig. 15, showing the temperature dependence of the 0–0 emission peak energy) are associated with two values of the energetic disorder. This phenomenon is connected with the packing of molecules in the sample, such as poly-domain structure, amorphous regions, or different energetic contributions from the surrounding molecules, as shown in Supplementary Fig. 18a (several peaks for ITIC molecules).

## Discussion

Exciton transport remains a bottleneck for high-efficiency organic photovoltaics. Thus, understanding the structure–property relationship(s) may lead to design rules that can aid the synthesis of improved materials, ultimately leading to OPVs with improved performance. By measuring the exciton diffusion length in a wide range of best-in-class NFA molecules in combination with quantum chemical simulations, we were able to identify key properties on a molecular level that explain the long diffusion length (up to 47 nm) measured. This long-range exciton transport appears to underpin the tremendous success of this new generation of NFAs in carrier photogeneration and extraction leading to the record efficiencies reported recently^6,9,10^. Overall, our findings can be rationalized to three design rules that can aid the synthesis of new materials with long exciton diffusion lengths: (i) increase the stiffness of the conjugated core; (ii) engineer the end-group substituents for favorable crystal packing; and (iii) increase the coupling between donor and acceptor blocks.

## Methods

### Materials

IT-2Cl (3,9-bis(2-methylene-((3-(1,1-dicyanomethylene)-chloro)-indanone))−5,5,11,11-tetrakis(4-hexylphenyl)-dithieno[2,3-d:2′,3′-d′]-s-indaceno[1,2-b:5,6-b′]dithiophene), IT-4F (3,9-bis(2-methylene-((3-(1,1-dicyanomethylene)-6,7-difluoro)-indanone))-5,5,11,11-tetrakis(4-hexylphenyl)-dithieno[2,3-d:2′,3′-d′]-s-indaceno[1,2-b:5,6-b′]dithiophene), and Y6 ((2,20-((2Z,20 Z)-((12,13-bis(2-ethylhexyl)-3,9-diundecyl-12,13-dihydro-[1,2,5] thiadiazolo[3,4-e] thieno[2,″30′:4′,50] thieno[20,30: 4,5]pyrrolo[3,2-g] thieno[20,30:4,5] thieno[3,2-b]indole-2,10-diyl)bis(methanylylidene))bis(5,6-difluoro-3-oxo-2,3-dihydro-1H-indene-2,1-diylidene))dimalononitrile)) were purchased from Solarmer Materials Inc. (Beijing). IT-M (3,9-bis(2-methylene-((3-(1,1-dicyanomethylene)-6/7-methyl)-indanone))-5,5,11,11-tetrakis(4-hexylphenyl)-dithieno[2,3-d:2′,3′-d′]-s-indaceno[1,2-b:5,6-b′]dithiophene), ITIC (3,9‐bis(2‐methylene‐(3‐(1,1‐dicyanomethylene)‐indanone)‐5,5,11,11‐tetrakis(4‐hexylphenyl)‐dithieno[2,3‐d:2′,3′‐d′]‐s‐indaceno[1,2‐b:5,6‐b′]‐dithiophene), IDIC (indacenodithiophene end capped with 1,1-dicyanomethylene-3-indanone), SF-PDI_2_ (spirobifluorene perylenediimide), and Phen-NaDPO (DPO)^34^ ((2‐(1,10‐phenanthrolin‐3‐yl)naphth‐6‐yl)diphenylphosphine oxide) were purchased from 1-Material Inc. PC_71_BM ([6,6]-Phenyl-C71-butyric acid methyl ester) was obtained from Solenne BV. EH-IDTBR (2-ethylhexyl rhodanine-benzothiadiazole-coupled indacenodithiophene) was synthesized at KAUST. All materials above were used as received. PESA measurements were recorded using a Riken Keiki PESA spectrometer (Model AC-2) with a power setting of 10 nW and a power number of 0.3. Samples for PESA were prepared on glass substrates.

### Thin-film preparation and solar cell fabrication

Indium tin oxide (ITO)-coated glass substrates (Kintec Company, 10 Ω/sq.) were cleaned by sequential ultra-sonication in dilute Extran 300 detergent solution, deionized water, acetone, and isopropyl alcohol for 20 min each. These substrates were then cleaned by UV–ozone treatment for 20 min. Copper (I) thiocyanate (CuSCN) (25 mg/ml) (Sigma-Aldrich) was dissolved in diethyl sulfide (DES) (Sigma-Aldrich) at 60 °C for 1 h and then filtered. The CuSCN solution was then spin-cast at 2500 rpm for 30 s, followed by annealing of the device at 105 °C for 10 min.

For bilayer CuSCN/acceptor devices, the acceptors were dissolved in chlorobenzene (CB) at different concentration (7–30 mg/ml) for EH-IDTBR, IT-M, ITIC, IT-2Cl, IT-4F, and PC_71_BM; or in chloroform (CF, 3–20 mg/ml) for Y6, IDIC, and SF-PDI_2_. The acceptor layers were spun on CuSCN layer at different speed for 30 s to obtain acceptor film with different thicknesses (5‒150 nm, film thicknesses were measured by using the Tencor surface profiler). A layer of 5 nm of Phen-NaDPO (DPO) as electron-transport layer (ETL) and exciton blocking layer (EBL) was spun from methanol solution (0.5 mg/ml) on top of the acceptor layer. Next, the samples were placed in a thermal evaporator for evaporation of a 100 nm-thick layer of aluminum evaporated at 5 Å s^−1^; the pressure of <2 × 10^-6^ Torr. Effective area of the tested solar cells is 0.1 cm^2^. This effective area was determined from the layout of ITO substrate and top contact mask.

Neat films of acceptors for optical measurements (UV–vis, PL, ellipsometry, TA) were prepared from either CB (20 mg/ml) or CF (10 mg/ml) and spin-coated onto quartz substrates (or Si/SiO_2_ substrate for GIWAXS, and ellipsometry measurements) in glovebox at 2000 rpm. To measure the intrinsic monomolecular decay constant from the dilute acceptors using TA, the blend film of acceptor with polystyrene was prepared from CF solution (2 mg/ml) and polystyrene (56 mg/ml) and mixed in 1:1 ratio.

### Solar cells characterizations and analysis

*J*–*V* measurements of solar cells were performed in a nitrogen-filled glove box using a Keithley 2400 source meter and an Oriel Sol3A Class AAA solar simulator calibrated to 1 sun, AM1.5 G, with a KG-5 silicon reference cell certified by Newport. The *J*–*V* curves were measured in the reverse direction, that is, from positive bias, with dwell time is 10 ms and 100 data points are collected. EQE was characterized using an EQE system (PV measurement Inc.). Measurements were performed at zero bias by illuminating the device with monochromatic light supplied from a Xenon arc lamp in combination with a dual-grating monochromator. The number of photons incident on the sample was calculated for each wavelength by using a silicon photodiode calibrated by The National Institute of Standards and Technology (NIST).

The cross-section TEM was conducted on a complete bilayer CuSCN/IT-4F devices to check the interface between the CuSCN and IT-4F layers. The lamellae were prepared using a focused ion beam (FIB) in a scanning electron microscope (Helios 400s, FEI) equipped with a nanomanipulator (Omniprobe, AutoProbe300). Sequential layers of carbon and platinum were deposited under electron and ion beams to protect the samples during the later stages of lamella preparation. Ga ion beam was used at 30 kV and 9 nA to mill the sample and then cut the lamella from the bulk. The lamella was mounted on the special TEM copper grid with the help of the nanomanipulator using the lift-out method. The lamella was then thinned down with the ion beam (30 kV, 93 pA) and cleaned from the contamination at lower voltages (5 kV, 48 pA). The lamellae were then imaged with the TEM (Titan 80-300, FEI) at the operating voltage of 300 kV.

### Thin-film transistor and time-resolved microwave conductivity

TFT device fabrication of the NFAs was following our previous procedure and details can be found in ref. ^33^. The electrical characterization of the transistors was carried out at room temperature in a nitrogen-filled glove box using an Agilent B2902A parameter analyzer.

TRMC measurements were performed using a system described in a previous report^62^. The measurements were carried out on bilayer CuSCN/NFA films, which were deposited on 0.9 cm × 0.9 cm quartz substrates. All the films were encapsulated with CYTOP. Films were measured in air at room temperature. The laser employed had a wavelength of 532 nm and a repetition rate of 15 Hz. The microwave frequency was ~8.5 GHz. The TRMC figure of merit $\phi {\mathrm{{\Sigma}}}\mu _{{\mathrm{TRMC}}} = \phi \left( {\mu _{\rm{e}} + \mu _{\rm{h}}} \right)$, served as a proxy for the sum of electron ($\mu _{\rm{e}}$) mobility and hole ($\mu _{\rm{h}}$) mobility, where $\phi$ is the fractional of electron–hole-pairs generated per absorbed photon. A representative value of $\phi {\mathrm{{\Sigma}}}\mu$ was evaluated for each sample by fitting fluence-dependent data to model accounting for bimolecular and Auger recombination^63^.

### Steady-state optical spectroscopy

The UV–vis absorption spectra of CuSCN and acceptor films were recorded using Cary 5000 spectrophotometer in the range 250–1100 nm. Fluoromax-4 spectrofluorometer from HORIBA Scientific was used to collect the steady-state PL. Temperature-dependent PL data of neat acceptors were recorded using LabRam Aramis Raman spectrometer from Horiba Jobin Yvon following 633 nm (for EH-IDTBR, Y6, IT-M, ITIC, IT-2Cl, IT-4F, and IDIC) and 473 nm (for PC_71_BM, and SF-PDI_2_) laser excitations. The PL quantum yield measurements of the acceptor films were carried out using an Edinburgh Instruments integrating sphere with an FLS920-s fluorescence spectrometer. The optical constants *n* and *k* for the active layers were collected by variable angle spectroscopic ellipsometry (VASE) with an M-2000 ellipsometer (J.A. Woolam Co., Inc). The VASE measurements were performed with incident angles being varied from 50° to 75° in steps of 5° relative to the samples. Transfer matrix modeling was used to simulate the maximum theoretical *J*_SC_ plots as a function of active layer thickness (thickness range: 0–200 nm); the model assumes 100% internal quantum efficiency (IQE). The transfer matrix code for these simulations was developed by George F. Burkhard and Eric T. Hoke; code available from: transfermatrix/index.html.

### Grazing incidence wide-angle X-ray scattering (GIWAXS)

GIWAXS measurements were performed at the Advance Light Source (ALS) (beamline 7.3.3); samples were prepared by following the same processing protocols as device fabrication using Si/SiO_2_ substrates. 10-keV X-ray beam was incident at a grazing angle of 0.13°, selected to maximize the scattering intensity from the samples, and the Dectris Pilatus 2M photon counting detector was used to detect the scattered X-rays, giving 2D GIWAXS patterns.

### Transient absorption (TA) spectroscopy

TA spectroscopy was carried out using a home-built pump–probe setup^64^. The output of a titanium:sapphire amplifier (Coherent LEGEND DUO, 4.5 mJ, 3 kHz, 100 fs) was split into three beams (2, 1, and 1.5 mJ). Two of them were used to separately pump two optical parametric amplifiers (OPA) (Light Conversion TOPAS Prime). The photophysical processes in this experiment were initiated by an ultrafast laser pulse generated by TOPAS 1 and were probed by broad white light supercontinuum which was generated by calcium fluoride crystal upon excitation with 1300 nm signal from TOPAS 2. Further details can be found elsewhere^64^. The nonfullerene acceptor (NFA) films were excited at different wavelengths: IDIC (600 nm), Y6 (700 nm), IT-M (700 nm), IT-4F (750 nm), IT-2Cl (700 nm), and ITIC (700 nm).

### Computational details

We extracted closely arranged dimer geometries of IDTBR, IT-4F, and IT-2Cl from their crystal structures. For ITIC and IT-M, where the crystal structures are not available but their molecular formulas are similar to IT-4F, we formed their dimer structures by using IT-4F dimer structure and substitute F atoms with H atoms and CH_3_ groups, respectively. To simplify the calculations, we substituted all side alkyl chains of the dimers with CH_3_ groups. The positions of H atoms and the CH_3_ groups were optimized at the m062×/6-311g(d,p)^65–67^ level, while other backbone atoms were kept frozen in order to retain the relative position between the two monomers.

Excited state and the follow-up diabatization calculations were performed with the time-dependent density-functional theory (DFT) method at the m062×/6-311g(d,p) level. For diabatization calculations, we define each molecule as a domain, and used up to eight excited states for constructing diabatic states. The coupling matrix of each dimer system consists of at least two local excitations (LEs) and two charge transfer (CT) states, respectively, which ensures a qualitatively correct coupling picture.

## Supplementary information

Supplementary Information File

## Data Availability

The source data are available online at ‘10.6084/m9.figshare.12871736’. Extra data are available from the corresponding authors upon request.

## Code availability

The codes or algorithms used to analyze the data reported in this study are available from the corresponding authors upon reasonable request.
