# Supplementary material for: Long-range exciton diffusion in molecular non-fullerene acceptors
Source: Nat Commun. 2020 Oct 15;11:5220. doi: 10.1038/s41467-020-19029-9 (PMC7562871; doi:10.1038/s41467-020-19029-9)
Supplement: Supplementary file 1 — Supplementary Information File [file 41467_2020_19029_MOESM1_ESM.docx]

# **Supplementary Information**

# **for**

# **Long-range exciton diffusion in molecular non-fullerene acceptors**

# Yuliar Firdaus,^1‡^ Vincent M. Le Corre,^2‡^ Safakath Karuthedath,^1^ Wenlan Liu,^3^ Anastasia Markina,^3^ Wentao Huang,^4^ Shirsopratim Chattopadhyay,^5^ Masrur Morshed Nahid,^6^ Mohamad I. Nugraha,^1^ Yuanbao Lin,^1^ Akmaral Seitkhan,^1^ Aniruddha Basu,^1^ Weimin Zhang,^1^ Iain McCulloch, ^1^ Harald Ade,^6^ John Labram,^5^ Frédéric Laquai,^1^ Denis Andrienko,^3^ L. Jan Anton Koster,^2*^ Thomas D. Anthopoulos^1*^

‡ These authors contributed equally.

Corresponding authors: [thomas.anthopoulos@kaust.edu.sa](mailto:thomas.anthopoulos@kaust.edu.sa); [l.j.a.koster@rug.nl](mailto:l.j.a.koster@rug.nl)

**Supplementary Tables**

**Supplementary Table 1**. Summary of optimized solar cell performance of bilayer CuSCN/acceptor cells. All cells were tested under standard illumination of AM 1.5G (100 mW cm^-2^). Number of solar cells tested > 10 cells.

| **Acceptor** | **V_OC_**  **(V)** | **J_SC_**  **(mA cm^-2^)** | **FF**  **(%)** | **PCE_avg_ (PCE_max_)**  **(%)** |
| --- | --- | --- | --- | --- |
| PC_71_BM | 0.92 ± 0.027 | 1.35 ± 0.11 | 40.1 ± 2.57 | 0.50 ± 0.029 (0.56) |
| EH-IDTBR | 0.76 ± 0.041 | 1.35 ± 0.06 | 28.7 ± 0.27 | 0.29 ± 0.01 (0.31) |
| SF-PDI_2_ | 1.02 ± 0.075 | 0.52 ± 0.02 | 32.2 ± 0.96 | 0.15 ± 0.037 (0.18) |
| IDIC | 0.86 ± 0.011 | 4.16 ± 0.13 | 43.7 ± 1.82 | 1.56 ± 0.089 (1.72) |
| ITIC | 0.92 ± 0.008 | 3.30 ± 0.14 | 37.9 ± 1.77 | 1.15 ± 0.092 (1.30) |
| IT-M | 0.89 ± 0.039 | 3.17 ± 0.14 | 33.7 ± 1.51 | 0.95 ± 0.102 (1.07) |
| Y6 | 0.82 ± 0.009 | 4.48 ± 0.09 | 51.7 ± 0.83 | 1.90 ± 0.059 (2.00) |
| IT-2Cl | 0.83 ± 0.011 | 4.49 ± 0.16 | 41.2 ± 1.81 | 1.54 ± 0.094 (1.74) |
| IT-4F | 0.81 ± 0.009 | 5.29 ± 0.21 | 57.3 ± 2.31 | 2.45 ± 0.099 (2.65) |

**Supplementary Table 2***.* Summary of the charge mobility values for acceptors measured by TFT and TRMC in this work. The TRMC figure of merit is the product of the sum of electron and hole mobilities and the carrier generation efficiency.

| **NFA** | **𝜇_electron,TFT_**  **(cm^2^V^-1^s^-1^)** | **𝜇_hole,TFT_**  **(cm^2^V^-1^s^-1^)** | **φΣμ_TRMC_**  **(cm^2^V^-1^s^-1^)** |
| --- | --- | --- | --- |
| PC_71_BM | 0.120 | 0.090 | 0.0013 |
| EH-IDTBR | - | - | 0.0014 |
| SF-PDI_2_ | - | - | 0.0113 |
| IDIC | 0.026 | 0.008 | 0.0012 |
| ITIC | 0.021 | 0.008 | 0.0020 |
| IT-M | - | - | 0.0094 |
| Y6 | 0.004 | 0.05 | 0.0095 |
| IT-2Cl | 0.025 | 0.005 | 0.0038 |
| IT-4F | 0.008 | 0.002 | 0.0012 |

**Supplementary Table 3***.* Parameters used in the drift-diffusion modeling for a CuSCN/IT-4F like bilayer solar cell. Further details on the drift-diffusion simulatin methods can be found in Ref. 27.

| **Parameters** | **Values** |
| --- | --- |
| Grid spacing | 1 nm |
| µ | 10^-4^/10^-2^ cm^2^/(Vs) |
| ε_r_ | 3.5 |
| γ_pre_ | 0.01/1 |
| N_cv_ | 10^19^ cm^-3^ |
| NFA L_D_ | 45 nm |
| WF_anode_ | 5.4 |
| WF_cathode_ | 4.24 |

**Supplementary Table 4***.* Calculation of exciton diffusion coefficient and diffusion length from SSA measurements with two different *R*.

| **NFA** | ***τ*** | ***α*** | ***R* (nm)** | | ***D* (10^-2^ cm^2^s^-1^)** | | ***L*_D_ (nm)** | |
| --- | --- | --- | --- | --- | --- | --- | --- | --- |
|  | **(ps)** | **(10^-7^ cm^3^s^-1^)** | ***R*_1_** | ***R*_2_** | ***D*_1_** | ***D*_2_** | ***L*_D,1_** | ***L*_D,2_** |
| IDIC | 241±8 | 0.28±0.003 | 1.0 | 1.57 | 1.1±0.011 | 0.7±0.008 | 16.4±0.29 | 13.1±0.23 |
| ITIC | 394±15 | 0.65±0.016 | 1.0 | 1.68 | 2.6±0.064 | 1.5±0.038 | 31.9±0.72 | 24.6±0.56 |
| IT-M | 392±8 | 0.78±0.020 | 1.0 | 1.88 | 3.1±0.080 | 1.7±0.042 | 34.9±0.57 | 25.4±0.42 |
| Y6 | 253±15 | 1.36±0.012 | 1.0 | 1.50 | 5.4±0.048 | 3.6±0.032 | 37.0±1.10 | 30.2±0.91 |
| IT-2Cl | 402±9 | 1.07±0.008 | 1.0 | 1.61 | 4.3±0.032 | 2.6±0.020 | 41.4±0.49 | 32.6±0.38 |
| IT-4F | 351±12 | 1.61±0.020 | 1.0 | 1.65 | 6.4±0.080 | 3.9±0.048 | 47.4±0.86 | 36.9±0.67 |

*R_2_ is d_100_-spacing of the NFAs with values were obtained from GIWAXS studies of neat NFAs.^1-6^*

**Supplementary Table 5***.* Measured values of singlet exciton diffusion length (*L*_D_ vs year plot is shown in **Fig. 4e**) and diffusion coefficient.

| **Material** | **Year** | ***L*_D_ (nm)** | ***D* (cm^2^ s^-1^)** | **Method** | **Ref** |
| --- | --- | --- | --- | --- | --- |
| **Polymer Donor** |  |  |  |  |  |
| NRS-PPV | 2005 | 6 | - | PL quenching in bilayer | ^7^ |
| MDMO-PPV | 2006 | 6 | - | PL quenching in bilayer | ^8^ |
| C-PCPDTBT | 2012 | 10.6 | 0.0026 | PL quenching in blends | ^9^ |
| Si-PCPDTBT | 2012 | 10.5 | 0.0011 | PL quenching in blends | ^9^ |
| P3HT | 2010 | 27±12 | 0.01 | TRPL | ^10^ |
| P3HT | 2012 | 5.4 | 0.00022 | PL quenching in blends | ^9^ |
| PBTTT | 2012 | 10 | - | Photocurrent | ^11^ |
| TFB | 2013 | 9 | - | PL quenching in blends | ^12^ |
| RRa-P3HT | 2015 | 14 | 0.0033 | Transient absorption | ^13^ |
| RR-P3HT-L | 2015 | 4.8 | 0.00046 | Transient absorption | ^13^ |
| RR-P3HT-H | 2015 | 20 | 0.0079 | Transient absorption | ^13^ |
| PBDB-T | 2018 | 10 | - | Photocurrent | ^14^ |
| PCDTBT | 2018 | 4 | - | PL quenching in blends | ^15^ |
| P3HT | 2018 | 6 | - | PL quenching in blends | ^15^ |
| PffBT4T-2OD | 2018 | 10.9 | 0.0021 | Transient absorption | ^16^ |
| PTB7 | 2019 | 4.5 | - | Photocurrent | ^17^ |
| **SM donor** |  |  |  |  |  |
| CuPc | 2007 | 15.4 | - | Photocurrent | ^18^ |
| H2Pc | 2007 | 11.9 | - | Photocurrent | ^18^ |
| FePc | 2007 | 1 | - | Photocurrent | ^18^ |
| CoPc | 2007 | 1.6 | - | Photocurrent | ^18^ |
| NiPc | 2007 | 9 | - | Photocurrent | ^18^ |
| ZnPc | 2007 | 15 | - | Photocurrent | ^18^ |
| NPD | 2010 | 5.1 | - | PL Quenching in trilayer | ^19^ |
| SubPc | 2010 | 7.7 | - | PL Quenching in trilayer | ^19^ |
| CBP | 2010 | 16.8 | - | PL Quenching in trilayer | ^19^ |
| ZnPc | 2017 | 10.1 | - | Photocurrent | ^20^ |
| DTS(FBTTH_2_)_2_ | 2017 | 15 | 0.0036 | TRPL | ^21^ |
| DR3TBDTT | 2019 | 15.7 | 0.0035 | TRPL | ^22^ |
| BTR | 2019 | 40 | 0.0040 | TRPL | ^23^ |
| BQR | 2019 | 43 | 0.0050 | TRPL | ^23^ |
| **Fullerene acceptor** |  |  |  |  |  |
| PC_61_BM | 2009 | 5 | - | TRPL | ^22^ |
| PC_71_BM | 2018 | 4.5 | - | PL Quenching in blends | ^15^ |
| PC_71_BM | 2013 | 3.1 | 0.00016 | PL Quenching in blends | ^24^ |
| C_60_ | 2019 | 21.3 | - | Photocurrent | ^17^ |
| C_70_ | 2019 | 7.4 | - | Photocurrent | ^17^ |
| **NFA** |  |  |  |  |  |
| FBR | 2018 | 5.5 | 0.0058 | Transient absorption | ^16^ |
| IDIC | 2019 | 35 | 0.02 | Transient absorption | ^25^ |

**Supplementary Table 6**. Summary of energetic disorder σ obtained from temperature dependent PL and Forster resonance energy transfer (FRET) parameters. *R*_0_ was calculated using dipole orientation factor (*κ*^2^ = 0.476) for fixed and randomly oriented dipoles.^26^

| **NFA** | ***σ*^Ϯ^**  **(meV)** | ***M*_W_**  **(g·mol^-1^)** | **Density**  **(g·cm^-3^)** | ***M*_W_**  **(g·mol^-1^)** | **Conc**  **(M)** | ***J***  **(10^30^ nm^6^·mol^-1^)** | ***ϕ*_PL_**  **(%)** | ***n*** | ***R*_0_**  **(nm)** |
| --- | --- | --- | --- | --- | --- | --- | --- | --- | --- |
| PC_71_BM | 15 | 1031 | 1.63 | 1031 | 1.581 | 0.15 | 0.1^*^ | 2.0 | 1.3 |
| EH-IDTBR | 48 | 1326 | 1.08 | 1326 | 0.814 | 1.11 | 2.0 | 1.85 | 3.1 |
| SF-PDI_2_ | 59 | 1822 | 1.50 | 1822 | 0.823 | 0.02 | 30.0 | 1.75 | 2.6 |
| IDIC | 34 | 1011 | 1.08 | 1011 | 1.068 | 1.00 | 1.0 | 1.83 | 2.7 |
| ITIC | 53 | 1428 | 1.08 | 1428 | 0.756 | 1.94 | 1.0 | 1.72 | 3.2 |
| IT-M | 59 | 1456 | 1.08 | 1456 | 0.742 | 1.90 | 1.0 | 1.84 | 3.0 |
| Y6 | 56 | 1452 | 1.08 | 1452 | 0.744 | 3.92 | 0.2 | 1.83 | 2.6 |
| IT-2Cl | 45 | 1495 | 1.08 | 1495 | 0.722 | 2.39 | 0.4 | 1.81 | 2.7 |
| IT-4F | 39 | 1500 | 1.08 | 1500 | 0.720 | 2.30 | 0.3 | 1.70 | 2.7 |

**Supplementary Table 7***.* Reorganization energy (*λ*) of selected NFA molecules along with their oscillator strength at the optimized ground state (*f*_gs_) and first excited state (*f*_ex_) geometries.

| **NFA** | $\boldsymbol{\lambda}$**(eV)** | ***f*_gs_** | ***f*_ex_** |
| --- | --- | --- | --- |
| EH-IDTBR | 0.396 | 3.040 | 2.750 |
| SF-PDI_2_ | - | 0.277 | ~0.3 |
| IDIC | 0.276 | 2.769 | 2.663 |
| ITIC | 0.292 | 3.208 | 2.961 |
| IT-M | 0.241 | 2.769 | 3.025 |
| Y6 | 0.242 | 2.483 | 2.337 |
| IT-2Cl | 0.281 | 3.221 | 3.038 |
| IT-4F | 0.281 | 3.154 | 2.973 |
| IT-4Cl | 0.274 | 3.276 | 3.099 |
| IT-DM | 0.307 | 3.253 | 3.076 |
| IT-2F | 0.280 | 3.123 | 2.937 |
| IEICO | 0.365 | 3.301 | 3.495 |
| IEICO-4Cl | 0.236 | 3.298 | 3.531 |
| IEICO-4F | 0.351 | 3.309 | 3.510 |

**Supplementary Table 8.** Charge transfer character weight (CT%) of calculated NFA dimer excited states.

| # state | IDTBR | ITIC | ITIC-2Cl-1 | ITIC-2Cl-2 | ITIC-4F |
| --- | --- | --- | --- | --- | --- |
| 1 | 0.000 | 0.009 | 0.009 | 0.012 | 0.008 |
| 2 | 0.000 | 0.009 | 0.010 | 0.015 | 0.008 |
| 3 | 0.000 | 0.052 | 0.063 | 0.029 | 0.041 |
| 4 | 0.000 | 0.019 | 0.018 | 0.039 | 0.028 |
| 5 | 1.000 | 0.954 | 0.954 | 0.807 | 0.944 |
| 6 | 1.000 | 0.926 | 0.915 | 0.885 | 0.938 |
| 7 | 0.000 | 0.013 | 0.319 | 0.405 | 0.286 |
| 8 | 0.003 | 0.056 | 0.012 | 0.232 | 0.011 |

**Supplementary Figures**

**Supplementary Figure 1***.* **Photoelectron spectroscopy in air (PESA) measurements for organic-acceptor and neat CuSCN films**. **a**, CuSCN, **b**, PC_71_BM. **c**, EH-IDTBR. **d**, SF-PDI_2_. **e**, IDIC. **f**, ITIC. **g**, IT-M. **h**, Y6. **i**, IT-2Cl. **j**, IT-4F. PESA-inferred ionization energies (IEs) are reported on the plots and in Fig. 1c.

**Supplementary Figure 2***.* **Thin-film transistor** (**TFT) measurements of various NFAs**. **a**, Device architecture used for the TFT measurement of NFA films. **b**, Transfer characteristics of IEICO. **c**, ITIC. **d**, IT-2Cl. **e**, IT-4F. **f**, IDIC. TG-BC transistors employing CYTOP as the dielectric, at various source-drain voltages. The channel lengths (L) and widths (W) of the transistors are 30 µm and 1 mm, respectively.

**Supplementary Figure 3**. **Grazing-Incidence Wide-Angle X-ray Scattering (GIWAXS) characterization**. GIWAXS 1D line profiles in the in-plane and out-of-plane directions for (a) the reference, CuSCN and NFA films: (b) SF-PDI_2_, (c) IDIC, (d) ITIC, (e) IT-M, (f) Y6, (g) IT-2Cl, and (h) IT-4F on Si/SiO_2_ substrate.

**Supplementary Figure 4***.* **Absorption and photoluminescence (PL) spectra of CuSCN and SF-PDI_2_ layers**. For comparison, absorption and PL spectra of SF-PDI_2_ film is also shown.

**Supplementary Figure 5***.* **Simulated evolution of the photocurrent density and device parameters for different layer thicknesses**. **a**, depending on *L*_D_ shows that it influences the magnitude of the photocurrent. **b**, More importantly, it strongly influences the shape of the normalized EQE vs thickness by setting its maximum and the decay trend at high thicknesses. Hence fitting this curve is the right approach to get the best possible estimation of *L*_D_. **c**, Fill-factors (FF) for different light intensities as a function of IT-4F thickness (from CuSCN/IT-4F devices). The difference in FF between the data obtained at 20.8 mW cm^−2^ to FF of 70% (grey area) is attributed to losses by geminate recombination (GR), and the difference between the data at 20.8 mW cm^−2^ and the data obtained at higher intensities ~100 mW cm^−2^ is attributed to nongeminate recombination (NGR). **d**, The J-V characteristics of CuSCN/IT-4F devices for different thicknesses (measured at AM1.5G illumination). Coupled exciton diffusion and drift-diffusion simulation of the EQE for 10^-4^ **e**, and 10^-2^ cm^2^V^-1^s^-1^) **f**, showing that the mobility and recombination rate constant have no effect on the photocurrent measurement. Parameters used for the drift-diffusion simulation can be found in **Supplementary Table 7**.

**Supplementary Figure 6***.* **Evolution of external quantum efficiency (EQE) as a function of acceptor layer thickness and excitation wavelength, *λ*_exc_, for PC_71_BM**. EQE of CuSCN/PC_71_BM bilayer devices: **a**, 450 nm. **b**, 475 nm. **c**, 500 nm. **d**, 525 nm. The experimental data (circles) are fitted (solid lines) for all thickness. *L*_D_ obtained from the fitting: 10 nm. The dashed lines are the fits for *L*_D_ 5 and 15 nm.

**Supplementary Figure 7***.* **Evolution of external quantum efficiency (EQE) as a function of acceptor layer thickness and excitation wavelength, *λ*_exc_, for EH-IDTBR**. EQE of CuSCN/EH-IDTBR bilayer devices: **a**, 650 nm. **b**, 675 nm. **c**, 700 nm. **d**, 725 nm. The experimental data (circles) are fitted (solid lines) for all thickness. *L*_D_ obtained from the fitting: 15 nm*.* The dashed lines are the fits for *L*_D_ 10 and 20 nm.

**Supplementary Figure 8***.* **Evolution of external quantum efficiency (EQE) as a function of acceptor layer thickness and excitation wavelength, *λ*_exc_, for SF-PDI_2_**. EQE of CuSCN/SF-PDI_2_ bilayer devices: **a**, 455 nm. b, 505 nm. **c**, 530 nm. **d**, 555 nm. The experimental data (circles) are fitted (solid lines) for all thickness. *L*_D_ obtained from the fitting: 20 nm. The dashed lines are the fits for *L*_D_ 15 and 25 nm.

**Supplementary Figure 9***.* **Evolution of external quantum efficiency (EQE) as a function of acceptor layer thickness and excitation wavelength, *λ*_exc_, for IDIC**. EQE of CuSCN/IDIC bilayer devices : **a**, 600 nm. **b**, 630 nm. **c**, 660 nm. **d**, 690 nm. The experimental data (circles) are fitted (solid lines) for all thickness. *L*_D_ obtained from the fitting: 24 nm. The dashed lines are the fits for *L*_D_ 20 and 30 nm.

**Supplementary Figure 10***.* **Evolution of external quantum efficiency (EQE) as a function of acceptor layer thickness and excitation wavelength, *λ*_exc_, for ITIC**. EQE of CuSCN/ITIC bilayer devices : **a**, 600 nm. **b**, 630 nm. **c**, 660 nm. **d**, 690 nm. The experimental data (circles) are fitted (solid lines) for all thickness. *L*_D_ obtained from the fitting: 25 nm. The dashed lines are the fits for *L*_D_ 20 and 30 nm.

**Supplementary Figure 11***.* **Evolution of external quantum efficiency (EQE) as a function of acceptor layer thickness and excitation wavelength, *λ*_exc_, for IT-M**. EQE of CuSCN/IT-M bilayer devices : **a**, 630 nm. **b**, 650 nm. **c**, 680 nm. **d**, 700 nm. The experimental data (circles) are fitted (solid lines) for all thickness. *L*_D_ obtained from the fitting: 30 nm. The dashed lines are the fits for *L*_D_ 20 and 40 nm.

**Supplementary Figure 12***.* **Evolution of external quantum efficiency (EQE) as a function of acceptor layer thickness and excitation wavelength, *λ*_exc_, for Y6**. EQE of CuSCN/Y6 bilayer devices : **a**, 650 nm. **b**, 700 nm. **c**, 750 nm. **d**, 800 nm. The experimental data (circles) are fitted (solid lines) for all thickness. *L*_D_ obtained from the fitting: 35 nm. The dashed lines are the fits for *L*_D_ 25 and 45 nm.

**Supplementary Figure 13***.* **Evolution of external quantum efficiency (EQE) as a function of acceptor layer thickness and excitation wavelength, *λ*_exc_, for IT-2CI**. EQE of CuSCN/IT-2Cl bilayer devices : **a**, 650 nm. **b**, 675 nm **c**, 700 nm. **d**, 725 nm. The experimental data (circles) are fitted (solid lines) for all thickness. *L*_D_ obtained from the fitting: 40 nm. The dashed lines are the fits for *L*_D_ 30 and 50 nm.

**Supplementary Figure 14***.* **Evolution of external quantum efficiency (EQE) as a function of acceptor layer thickness and excitation wavelength, *λ*_exc_, for IT-4F**. EQE of CuSCN/IT-4F bilayer devices : **a**, 650 nm. **b**, 675 nm. **c**, 700 nm. **d**, 725 nm. The experimental data (circles) are fitted (solid lines) for all thickness. *L*_D_ obtained from the fitting: 45 nm. The dashed lines are the fits for *L*_D_ 35 and 55 nm.

**Supplementary Figure 15***.* **Temperature-dependent steady-state photoluminescence (PL) measurements.** PL spectra (top) and temperature dependence of 0-0 emission peak energy (bottom) of acceptor films: **a**, PC_71_BM. **b**, EH-IDTBR. **c**, SF-PDI_2_. **d**, IDIC. **e**, ITIC. **f**, IT-M. **g**, Y6. **h**, IT-2Cl. **i**, IT-4F.

**Supplementary Figure 16***.* **Optical properties of organic-acceptors**. Absorption coefficient and normalized PL of acceptor films (their bandgap values are also presented): **a**, PC_71_BM. **b**, EH-IDTBR. **c**, SF-PDI_2_. **d**, IDIC. **e**, ITIC. **f**, IT-M. **g**, Y6. **h**, IT-2Cl. **i**, IT-4F.

**Supplementary Figure 17**. **Exciton diffusion length and Förster radius**. Correlation between the measured exciton diffusion length and estimated cube of Förster radius (*R*_0_^3^) following the relation *L*_D_ ~ *R*_0_^3^*d*^-2^.^28^

**Supplementary Figure 18**. **Energetic disorder calculations and crystal structures of NFAs**. **a**, Histograms of the crystal field contribution to the energy of excited state in vacuum for IDTBR, IEICO-4F, ITIC-4F and ITIC. All distributions are normalized by total number of molecules in each system. **b**, Crystal structures of IEICO-4F and ITIC-4F.

**Supplementary References**

1 Sun, C. *et al.* A low cost and high performance polymer donor material for polymer solar cells. *Nat. Commun.* **9**, 743, doi:10.1038/s41467-018-03207-x (2018).

2 Gao, L. *et al.* High-Efficiency Nonfullerene Polymer Solar Cells with Medium Bandgap Polymer Donor and Narrow Bandgap Organic Semiconductor Acceptor. *Adv. Mater.* **28**, 8288-8295, doi:10.1002/adma.201601595 (2016).

3 Jiang, H. *et al.* Impact of the Siloxane-Terminated Side Chain on Photovoltaic Performances of the Dithienylbenzodithiophene–Difluorobenzotriazole-Based Wide Band Gap Polymer Donor in Non-Fullerene Polymer Solar Cells. *ACS Appl. Mater. Interfaces* **11**, 29094-29104, doi:10.1021/acsami.9b08722 (2019).

4 Yuan, J. *et al.* Single-Junction Organic Solar Cell with over 15% Efficiency Using Fused-Ring Acceptor with Electron-Deficient Core. *Joule* **3**, 1140-1151, doi:<https://doi.org/10.1016/j.joule.2019.01.004> (2019).

5 Zhang, H. *et al.* Over 14% Efficiency in Organic Solar Cells Enabled by Chlorinated Nonfullerene Small-Molecule Acceptors. *Adv. Mater.* **30**, 1800613, doi:10.1002/adma.201800613 (2018).

6 Chen, M. *et al.* Influences of Non-fullerene Acceptor Fluorination on Three-Dimensional Morphology and Photovoltaic Properties of Organic Solar Cells. *ACS Appl. Mater. Interfaces* **11**, 26194-26203, doi:10.1021/acsami.9b07317 (2019).

7 Markov, D. E., Tanase, C., Blom, P. W. M. & Wildeman, J. Simultaneous enhancement of charge transport and exciton diffusion in poly($p$-phenylene vinylene) derivatives. *Phys. Rev. B* **72**, 045217, doi:10.1103/PhysRevB.72.045217 (2005).

8 Scully, S. R. & McGehee, M. D. Effects of optical interference and energy transfer on exciton diffusion length measurements in organic semiconductors. *J. Appl. Phys.* **100**, 034907, doi:10.1063/1.2226687 (2006).

9 Mikhnenko, O. V. *et al.* Exciton diffusion length in narrow bandgap polymers. *Energy Environ. Sci.* **5**, 6960-6965, doi:10.1039/C2EE03466B (2012).

10 Cook, S., Liyuan, H., Furube, A. & Katoh, R. Singlet Annihilation in Films of Regioregular Poly(3-hexylthiophene): Estimates for Singlet Diffusion Lengths and the Correlation between Singlet Annihilation Rates and Spectral Relaxation. *The Journal of Physical Chemistry C* **114**, 10962-10968, doi:10.1021/jp101340b (2010).

11 Kozub, D. R. *et al.* Direct measurements of exciton diffusion length limitations on organic solar cell performance. *Chem. Commun.* **48**, 5859-5861, doi:10.1039/C2CC31925J (2012).

12 Bruno, A., Reynolds, L. X., Dyer-Smith, C., Nelson, J. & Haque, S. A. Determining the Exciton Diffusion Length in a Polyfluorene from Ultrafast Fluorescence Measurements of Polymer/Fullerene Blend Films. *The Journal of Physical Chemistry C* **117**, 19832-19838, doi:10.1021/jp404985q (2013).

13 Ohkita, H., Tamai, Y., Benten, H. & Ito, S. Transient Absorption Spectroscopy for Polymer Solar Cells. *IEEE J. Sel. Top. Quantum Electron.* **22**, 100-111, doi:10.1109/JSTQE.2015.2457615 (2016).

14 Zhang, J. *et al.* Efficient non-fullerene organic solar cells employing sequentially deposited donor–acceptor layers. *J. Mater. Chem.* **6**, 18225-18233, doi:10.1039/C8TA06860G (2018).

15 Zarrabi, N., Yazmaciyan, A., Meredith, P., Kassal, I. & Armin, A. Anomalous Exciton Quenching in Organic Semiconductors in the Low-Yield Limit. *The Journal of Physical Chemistry Letters* **9**, 6144-6148, doi:10.1021/acs.jpclett.8b02484 (2018).

16 Cha, H. *et al.* Influence of Blend Morphology and Energetics on Charge Separation and Recombination Dynamics in Organic Solar Cells Incorporating a Nonfullerene Acceptor. *Adv. Funct. Mater.* **28**, 1704389, doi:10.1002/adfm.201704389 (2018).

17 Zhang, T., Dement, D. B., Ferry, V. E. & Holmes, R. J. Intrinsic measurements of exciton transport in photovoltaic cells. *Nat. Commun.* **10**, 1156, doi:10.1038/s41467-019-09062-8 (2019).

18 Terao, Y., Sasabe, H. & Adachi, C. Correlation of hole mobility, exciton diffusion length, and solar cell characteristics in phthalocyanine/fullerene organic solar cells. *Appl. Phys. Lett.* **90**, 103515, doi:10.1063/1.2711525 (2007).

19 Luhman, W. A. & Holmes, R. J. Investigation of Energy Transfer in Organic Photovoltaic Cells and Impact on Exciton Diffusion Length Measurements. *Adv. Funct. Mater.* **21**, 764-771, doi:10.1002/adfm.201001928 (2011).

20 Siegmund, B. *et al.* Exciton Diffusion Length and Charge Extraction Yield in Organic Bilayer Solar Cells. *Adv. Mater.* **29**, 1604424, doi:10.1002/adma.201604424 (2017).

21 Long, Y. *et al.* Effect of Annealing on Exciton Diffusion in a High Performance Small Molecule Organic Photovoltaic Material. *ACS Appl. Mater. Interfaces* **9**, 14945-14952, doi:10.1021/acsami.6b16487 (2017).

22 Zhang, Y. *et al.* Enhanced exciton harvesting in a planar heterojunction organic photovoltaic device by solvent vapor annealing. *Org. Electron.* **70**, 162-166, doi:<https://doi.org/10.1016/j.orgel.2019.03.014> (2019).

23 Sajjad, M. T. *et al.* Tailoring exciton diffusion and domain size in photovoltaic small molecules by annealing. *J. Mater. Chem. C* **7**, 7922-7928, doi:10.1039/C9TC00951E (2019).

24 Hedley, G. J. *et al.* Determining the optimum morphology in high-performance polymer-fullerene organic photovoltaic cells. *Nat. Commun.* **4**, 2867, doi:10.1038/ncomms3867 (2013).

25 Chandrabose, S. *et al.* High Exciton Diffusion Coefficients in Fused Ring Electron Acceptor Films. *J. Am. Chem. Soc.* **141**, 6922-6929, doi:10.1021/jacs.8b12982 (2019).

26 Lin, J. D. A. *et al.* Systematic study of exciton diffusion length in organic semiconductors by six experimental methods. *Mater. Horiz.* **1**, 280-285, doi:10.1039/C3MH00089C (2014).

27 Koster, L. J. A. *et al.* Morphology and Efficiency: The Case of Polymer/ZnO Solar Cells. *Adv. Energy Mater.* **3**, 615-621, doi:10.1002/aenm.201200787 (2013).

28 Menke, S. M., Luhman, W. A. & Holmes, R. J. Tailored exciton diffusion in organic photovoltaic cells for enhanced power conversion efficiency. *Nat. Mater.* **12**, 152-157, doi:10.1038/nmat3467 (2013).
